# Telescoper: *de novo* assembly of highly repetitive regions

**DOI:** 10.1093/bioinformatics/bts399

**Published:** 2012-09-03

**Authors:** Ma'ayan Bresler, Sara Sheehan, Andrew H. Chan, Yun S. Song

**Affiliations:** ^1^Department of EECS; ^2^Department of Statistics, University of California, Berkeley, CA 94720, USA

## Abstract

**Motivation:** With advances in sequencing technology, it has become faster and cheaper to obtain short-read data from which to assemble genomes. Although there has been considerable progress in the field of genome assembly, producing high-quality *de novo* assemblies from short-reads remains challenging, primarily because of the complex repeat structures found in the genomes of most higher organisms. The telomeric regions of many genomes are particularly difficult to assemble, though much could be gained from the study of these regions, as their evolution has not been fully characterized and they have been linked to aging.

**Results:** In this article, we tackle the problem of assembling highly repetitive regions by developing a novel algorithm that iteratively extends long paths through a series of read-overlap graphs and evaluates them based on a statistical framework. Our algorithm, Telescoper, uses short- and long-insert libraries in an integrated way throughout the assembly process. Results on real and simulated data demonstrate that our approach can effectively resolve much of the complex repeat structures found in the telomeres of yeast genomes, especially when longer long-insert libraries are used.

**Availability:** Telescoper is publicly available for download at sourceforge.net/p/telescoper.

**Contact:** yss@eecs.berkeley.edu

**Supplementary Information:** Supplementary data are available at *Bioinformatics* online.

## 1 INTRODUCTION

Recent advances in high-throughput sequencing (HTS) technologies have dramatically reduced the cost of producing reference genomes for new species and characterizing whole-genome variations in multiple individuals of a population. However, the assemblies produced by current algorithms are often incomplete. Alkan *et al.* (2011) report that a *de novo* shotgun assembly of the human genome using short-reads is 16% shorter than the reference assembled using more laborious means, and that *<* 1% of segmental duplications are represented. Indeed, it is well recognized that there is room for better algorithmic use of the data, especially for repetitive regions, which are one of the primary challenges in assembly. Telomeres are particularly complex and repetitive, and thus very difficult to assemble correctly. Not only does each telomere contain repeats within itself, but often telomeres on different chromosomes are very similar. Existing assembly algorithms thus frequently fail to assemble telomeric regions from short-read data. Due to this lack of complete assembly, telomere evolution has not been fully characterized, though a great deal is to be gained from it, as telomeres have been linked to ageing (McEachern *et al.*, 2000). High-quality telomere assemblies could help us learn more about the variation in telomeres within and between species. In addition, characterizing telomere gene families and their regulation could help us clarify the function of telomeres and how they change as we age.

Genome assembly is the challenge of piecing together reads to reconstruct the original genome. Reads are obtained from various technologies, with varying read length, error rates and coverage. Sanger-chemistry reads range in length from around 500 to 1000 bases. Newer technologies such as Illumina, Complete Genomics (Drmanac *et al.*, 2010), Helicos (Harris *et al.*, 2008), 454 Life Sciences (Margulies *et al.*, 2005), SOLiD (McKernan *et al.*, 2009) and Ion Torrent (Rothberg *et al.*, 2011) provide reads at vastly lower costs for greater throughput, but at the expense of length. Initial improvements in assembly from short-read data focused on how to process the sheer quantity of data and how to detect overlaps. The de Bruijn graph proved a useful data structure for this purpose (Pevzner *et al.*, 2001) and is used by pioneering short-read assemblers such as Velvet (Zerbino and Birney, 2008) and EULER-USR (Chaisson *et al.*, 2009), and subsequent assemblers including SOAPdenovo (Li *et al.*, 2010), ALLPATHS 2 (MacCallum *et al.*, 2009), ABySS (Simpson *et al.*, 2009) and Cortex (Iqbal *et al.*, 2012).

Many HTS platforms produce paired-end or mate-pair reads, which we collectively refer to as read-pairs. The paired nature of these reads constitutes a powerful source of information, significantly facilitating genome assembly. Improved use of read-pair information lies at the heart of recent works such asALLPATHS-LG (Gnerre *et al.*, 2011), the PE-Assembler (Ariyaratne and Sung, 2011) and the Paired de Bruijn Graph (Medvedev *et al.*, 2011), innovations of which are primarily in earlier use of short-insert read-pairs.

ALLPATHS-LG requires reads of length around 100 bases sequenced from short fragments of length ≈ 180 bp so that, on average, each read-pair overlaps by ∼20 bases. This means that in general each read-pair can be merged into a single longer read corresponding to the fragment. A drawback of this approach is in the very specific type of data required, which differs from the standard library construction of fragments 300–500 bp in size. The PE-Assembler builds short stretches in non-repetitive regions first, similar to unitigs (see Section 2 for a definition) in a de Bruijn graph, and then extends these iteratively using reads with mates that map to the increasing already-assembled portion. (A similar idea is also used in Parrish *et al.* (2011) for resequencing with a reference, where insertions are assembled as iterative extensions of existing sequences.) The Paired de Bruijn Graph method entails building a so-called A-Bruijn graph in which vertices track pairs of reads instead of single reads, with two vertices being merged only if the merging is consistent with the associated pairs of reads. To our knowledge, this method remains largely theoretical at this time, and it has been tested only on simulated data with perfect reads.

A theoretical observation from Medvedev *et al.* (2011) is that longer long-insert libraries can substantially improve assembly. Recent innovations (Peng *et al.*, 2012) in library construction may bring such libraries into the mainstream, so it is timely to develop algorithms that take full advantage of such data.

In this article, we describe a new algorithm to improve *de novo* assembly of highly repetitive regions. Although the ideas presented here are applicable to the assembly of any genomic region, our algorithm was developed with the specific aim of assembling highly repetitive regions such as telomeres. In our method, which we name Telescoper, we incorporate the following three algorithmic ideas, the latter two of which make novel use of read-pairs:

1. **Iterative extensions:** a seed sequence is extended iteratively using reads localized to a particular region by their mates, thus allowing for gradual extension into difficult regions. See Section 3.1 for details. As mentioned above, this idea is not new, but it has not yet been fully exploited in a well-used algorithm, despite several potential advantages.
2. **Simultaneous use of short-insert read-pairs in a statistical framework:** rather than using read-pair information pair by pair to untangle the read-graph, we build extensions through the graph and *simultaneously* consider all read-pairs mapping to each extension to choose the most probable extension. See Section 3.2.
3. **Simultaneous use of long-insert libraries:** rather than using long-insert read-pairs only for scaffolding or for filling in gaps between easily assembled contigs, our iterative extension procedure uses long-insert reads during assembly. We look for support of assembled sequence at all insert sizes, so that incorrect assembly can result only if the repetitive structure spans all libraries. See Section 3.3 for further details.

Each of the above ideas helps to resolve repetitive regions. Implicit throughout our algorithm is the principle that in order to assemble difficult regions, one cannot make only safe simplifications, but must also explore several alternative extensions and use downstream analysis to find and reject false extensions.

We tested the performance of our method on both real and simulated data from the telomeres of the *Saccharomyces cerevisiae* genome, which consists of 16 chromosomes. This is a particularly challenging problem since all such telomeres have a core repetitive component called X (≈ 475 bp long) as well as several combinatorial repeats and sometimes a larger repetitive component (see Saccharomyces Genome Database, www.yeastgenome.org). In addition, because *S.cerevisiae* underwent an ancient genome duplication (Kellis *et al.*, 2004), telomeric regions of different chromosomes typically share highly similar repetitive regions. We show that Telescoper is capable of generating more complete and continuous assemblies in the telomeric regions than other state-of-the-art *de novo* assembly algorithms, especially when longer long-insert libraries are used.

## 2 TERMINOLOGY

We adopt the following terms commonly used to describe the output of sequencing technologies and the resulting assemblies:

- **Read-pair:** refers to a pair of sequenced reads from a fragment. The fragment size determines the distance between the two reads, often called the ‘insert’ size. The insert distribution is frequently approximated by a normal distribution. We use the term read-pair regardless of whether the insert is short or long.
- **Mate:** refers to the partner of a read *R* in a read-pair. When *R* is oriented with respect to a sequence, we know its mate's relative position and can refer to it as a ‘left-mate’ or ‘right-mate’ (or, as a ‘left-read’ or ‘right-read’).
- **Contig:** a sequence, which ideally belongs to the original genome, produced from assembling a group of reads. The standard output from an assembly algorithm is a set of contigs. Contigs are often ordered to produce ‘scaffolds’, which may contain stretches of unknown sequence between the contigs.
- **Read-overlap graph:** also called a read-graph, is a graph in which each vertex is a read and directed edges between reads represent overlapping sequence, i.e. in the error-free case, the last *k* bases of one read are the same as the first *k* bases of its neighbor read, where *k* is greater than some threshold.
- **Unitig:** a path through the read-graph that can be unambiguously merged into a single sequence. A ‘unitig graph’is an extension of the read-overlap graph idea (similarly for a unitig path), where the vertices are now unitigs.

## 3 METHODS

We have two main aims in our algorithm: (i) rather than performing a greedy read-by-read assembly procedure, we build a number of alternative extensions, and score them according to the alignment of read-pairs to each extension and (ii) we use long-insert read-pairs not only for scaffolding or gap filling but also as part of the assembly itself, to check that the local assembly is consistent on a longer scale.

Our algorithm begins with a set of non-repetitive ‘seed strings’, as could be taken from a reference genome, if it exists, or be assembled from a de Bruijn graph. At present, we use seeds of length 500 bp from the reference, at position 40 kb from the end of the chromosome, although contigs produced from any other algorithm could be used. The goal is to then independently extend each contig to produce a more complete assembly.

**Fig. 1.**
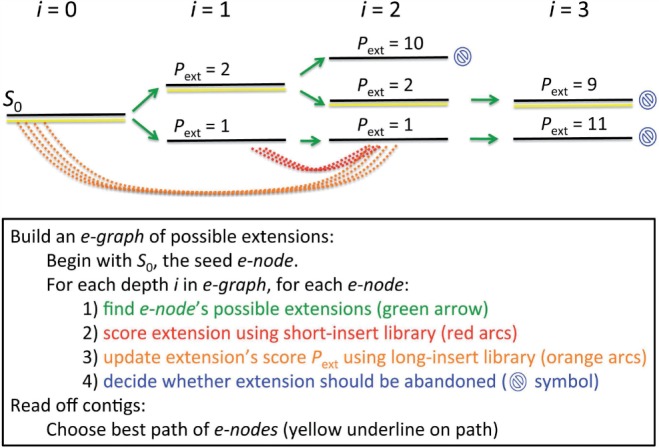
High-level description of the algorithm. Beginning with the seed string *S*_0_, the algorithm iteratively performs the steps described to construct an e-graph data structure, from which a contig or contigs can be read. For simplicity, only a few example arcs are shown; in reality, red arcs are present between each consecutive pair of e-nodes, and orange arcs can be present between a given e-node and any of its preceding e-nodes

A high-level overview of the algorithm is illustrated in Figure 1. The algorithm proceeds by extending the end of the contig iteratively by a fixed amount, *N*_new_, per iteration, as detailed in Section 3.1. We fix the extension length (usually a few hundred bases) as a conservative measure. Because multiple extensions are frequently possible, the result is an ‘extension graph’ (e-graph) in which each extension node (e-node) contains *N*_new_ bases of new sequence that serve as a possible extension for that e-node's parent. A path from the root (the seed string) to a leaf represents a series of extensions that form a single lengthened contig. The aim is for the e-graph to contain a path corresponding to the true sequence, ideally terminating close to the end of the desired chromosome, and for this path to be identifiable as the best.

Our algorithm will be most tractable if the e-graph is sparse, so at each iteration, there are as few extensions as possible (and the true extension is among them). The criteria for pruning and terminating the e-graph are discussed in Section 3.4. We first explain our methods for (i) listing possible extensions for a given e-node in the e-graph, (ii) scoring each extension based on the alignment of short-insert read-pairs and (iii) scoring each extension based on the alignment of long-insert read-pairs.

In the following description, we assume without loss of generality that we are extending to the right.

### 3.1 Iterative extension of assembly

The extension step consists of finding possible extensions of a given e-node; the extensions will in turn become e-nodes themselves. We fix the length of each e-node so that most right-reads in the new extension will have left-mates mapping to the e-node rather than behind it. In our implementation, we choose this length, denoted *N*_tot_, to be the mean insert length plus the standard deviation of the short-insert library. In the case of multiple short-insert libraries, one can use the largest short-insert length for computing *N*_tot_.

**Fig. 2.**
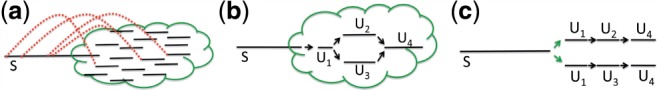
Illustration of Step 1 of Figure 1, finding an e-node *S*'s possible extensions. (**a**) A read ‘cloud’ consists of those right-reads with left-mates that map to *S*. (**b**) The reads in the cloud are then error corrected and organized into a read-graph, which is in turn converted into a unitig graph. (**c**) Paths through the unitig graph correspond to possible extensions

The extension step is depicted in Figure 2. It begins by mapping all the left-reads to the e-node to obtain right-mates extending off the right end of the e-node into unknown region yet to be assembled, i.e. the left-mate maps to the e-node and the right-mate dangles off the end, as illustrated in Figure 2a. We say that these right-mates form a read ‘cloud’.

The reads in the read cloud are error-corrected, then used to construct a read-overlap graph, which is transformed into a unitig graph as depicted in Figure 2b. More details on error-correction and read-overlap graph construction are provided in the Supplementary Material. The unitig graph encodes a list of candidate extensions for the contig, as illustrated in Figure 2c. Each new e-node consists of *N*_new_ bases of new extension plus (*N*_tot_ – *N*_new_) bases from the end of the previous e-node.

There are several advantages to this localized assembly. First, it reduces ambiguities caused by repeats. For a read-pair from another location to interfere with the area under construction, its left-read must map to the previous e-node while the right-read must overlap with another read in the read cloud. Second, because it restricts assembly to a small region, there is ample memory to store complicated information about the reads and their relationships. This information can be thrown out as we move to other regions of the graph. This local use of information enables more complex use of read-pairs, as described in Sections 3.2 and 3.3.

### 3.2 Simultaneous use of short-insert read-pairs in statistical scoring of extensions

Although existing assembly algorithms make use of read-pairs in various ways, the information contained in read-pairs has not yet been fully exploited. In other assemblers, read-pairs are used primarily to connect unitigs with expected insert sizes. We can obtain additional power by scoring potential extensions according to the features derived from the aligned read-pairs.

**Fig. 3.**
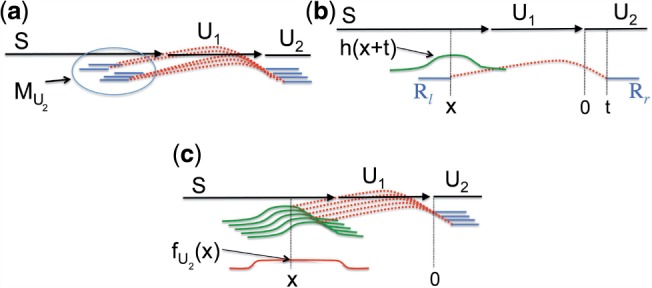
Computing the expected number of left-reads mapping back from a unitig *U*_2_ to the previous e-node *S*. (**a**) *M*_*U*_2__ denotes the set of reads mapping from unitig *U*_2_ to the previous e-node *S*. (**b**) For a right-read *R*_r_ located at position t in unitig *U*_2_, the probability of its left-mate *R_l_* mapping to *S* at a distance *x* behind *U*_2_ is *h*(*x* + *t*), where *h*(·) is the expected insert distribution. (**c**) The expected number of reads at position *x* behind unitig *U*_2_ is given by *f_U_* (*x*) defined in Equation (1)

We first evaluate extensions based on the likelihood of gaps in short-insert read-pair coverage. Each extension consists of an ordered sequence of unitigs, as in Figure 2c. Each right-read in an assembled unitig will have a left-mate mapping to earlier sequence in the previous e-node. The set of left-mates associated with reads in unitig *U* is denoted *M_U_* (Fig. 3a).

**Fig. 4.**
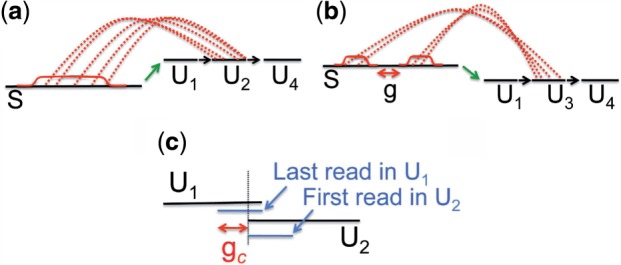
Illustration of Step 2 of Figure 1, scoring an e-node's possible extensions using short-insert read-pairs. (**a**) The penalty for unitig *U*_2_ is 0 because no gaps of size ≥*ℓ/*2 exist (where *ℓ* is the read length). (**b**) The penalty for unitig *U*_3_ is *>* 0 because a gap, denoted *g*, of size ≥ *ℓ/*2 exists. (**c**) The size of contig gap *g_c_* is the distance between the reads that define the end and start of two adjacent unitigs

In our model, we make the simplifying assumption of a uniform coverage distribution. Let *x* denote the distance from the right end of a left read relative to the start of unitig *U*, as pictured in Figure 3b. We denote by *f_U_* (*x*) the expected number of left-reads in *M_U_* spanning position *x* (Fig. 3c). We compute *f_U_* (*x*) by convolving the expected insert distribution *h*(·) with the uniform distribution over the stretch of *U* on which right mates can begin:
(1)
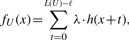

where *L*(*U*) is the length of *U*, *ℓ* is the read length and *λ* is the probability of a read arriving at position *t*; note that *λ* is equal to *C/*(2*ℓ*), where *C* is the coverage. False unitigs will typically have gaps in the empirical distribution 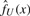, as illustrated in Figure 4b. Let Gap(*U*) denote the set of such gaps associated with *U* . For a gap *g* ∈ Gap(*U*) of length ≥ *ℓ/*2, we compute a penalty equal to the number of mates expected in *g*, obtained by summing *f_U_* (*x*) over *g*'s coordinates. The preliminary score for an extension is then the sum of these penalties over all gaps and all unitigs in the extension:
(2)
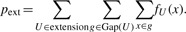

To produce a final score *P*_ext_ for each possible extension, we add *p*_ext_ to a ‘contig gap penalty’, equal to *λ* times the largest gap size (denoted by *g_c_* in Fig. 4c) between two adjacent unitigs, i.e. the expected number of reads to fall in that gap. The best extensions (i.e. those with the lowest *P*_ext_ scores) are kept, as described in more detail in Section 3.4.

### 3.3 Simultaneous use of long-insert libraries

Telescoper uses all libraries simultaneously during assembly, rather than using long-insert libraries only during scaffolding or gap-filling, as is typical in other assembly algorithms. The main idea is that once long paths have been formed in the e-graph, any further extension can be evaluated on the basis of its agreement with the current e-graph according to each library. Having produced and pruned a set of extensions using just the short-insert library in Steps 1 and 2 of our algorithm (Fig. 1), the third step aims to confirm that a proposed extension is supported by read-pair information from all other libraries simultaneously. For there to be ambiguity in extension choice, there must be repeats at lengths corresponding to all library sizes.

To test for long-insert read-pair support of a potential extension, we first gather all read-pairs of which right reads map to the extension and left-reads map to the previous e-nodes in the path up the e-graph. Then, if the right reads fully cover the proposed extension, even possibly without overlaps, we consider the extension to be fully supported. Partial support is computed as a linear function of the fraction of the extension that is covered by the right reads. This support measure is then multiplied by the short-insert score *P*_ext_ to obtain a single final score.

### 3.4 Choosing extensions for continuation

For a given e-node, upon finding all its possible extensions, at most *B* top scoring (the lower the better) extensions are retained for computational tractability. In our analysis, we use *B* = 4. We create a new e-node for each of these top scoring extensions and assign a running score equal to the sum of its extension score and its parent e-node's running score. Then, at each depth in the e-node graph, the *B* top scoring e-nodes are marked for pursuit.

An e-node is terminated if it cannot be lengthened by the extension operation, if its extension score plus the scores of two previous ancestral extensions exceeds a threshold, or if a specified maximum depth is reached.

To track the parallel success of alternative e-node paths and keep their number in check, we use breadth first search to explore the e-graph. If two different sequences of e-nodes end with equivalent e-nodes at a particular depth, we allow the two e-nodes to merge. This kind of merging of e-nodes reduces the computational burden.

## 4 EMPIRICAL RESULTS

In this section, we compare Telescoper's performance with that of other short-read assembly algorithms, including ABySS (Simpson *et al.*, 2009), ALLPATHS 2 (MacCallum *et al.*, 2009), SGA (Simpson and Durbin, 2012), SOAPdenovo (Li *et al.*, 2010) and Velvet (Zerbino and Birney, 2008).

Because of limited space, we focus on short-read data in the ensuing discussion. However, as detailed in the Supplementary Material, we also considered a combination of short-insert short-read data and long-insert Sanger read data, and observed that Telescoper compares favourably with other algorithms, including Celera (Myers *et al.*, 2000), which was designed for Sanger reads.

### 4.1 Data and experiment setup

We studied the performance on both simulated and real data from strain S288C of *S. cerevisiae*. We obtained a reference genome from Saccharomyces Genome Database (www.yeastgenome.org), which was created through extensive, systematic sequencing to produce a very accurate assembly, including the telomeric regions. As mentioned earlier, because of ancient genome duplication and complex yeast telomere structure, the telomeres of different chromosomes typically share highly similar repetitive regions, which poses challenges to assembly.

We considered different types of data to test the robustness of the algorithms and to study the effect of insert distributions on performance:

**Simulated Data D1** consisted of read-pairs with two insert distributions, one short and one long. The read length was 101 bp for both types. The *short-insert* reads had coverage depth 100× and an insert distribution with mean 400 bp and variance 75 bp. The *long-insert* reads had coverage depth 20× and an insert distribution with mean 10 kb and variance 1 kb. Simulation details are provided in the Supplementary Material.

**Simulated Data D2** consisted of two read-pair datasets with the same insert distributions and coverages as D1, but with a reduced read length of 50 bp.

**Real Data D3** consisted of Illumina read-pairs from a sequencing library preparation using Cre-Lox recombination. The reads, as described in Van Nieuwerburgh *et al.* (2012), were sorted using DeLoxer into reads categorized as *short-insert* (0–400 bp fragments, mean 220 bases) or *long-insert* (1–5 kb, mean 2.3 kb). The reads varied in length from 30–100 bp. We truncated reads to 50 bases in order to provide algorithms with high-quality, uniform-length reads. We used coverage 120× for the short-insert data and 40× for the long-insert data. The performance of Telescoper does not degrade with higher coverage data.

We sought to assess assembly for the 40-kb telomeric regions at the ends of each of *S. cerevisiae*'s 16 chromosomes. To this end, we simulated data only from this region. For the real data, we used the full dataset, but restricted evaluation statistics of the produced contigs to those alignable to the 32 telomeres, each of length 40 kb.

Details of running the various algorithms, including parameter settings and runtimes, can be found in the Supplementary Material. To optimize the performance of the other algorithms, insert distribution and coverage parameters were provided where appropriate. We did not include SGA for D2 and D3 since it was designed for reads of at least 100 bp.

### 4.2 Assembly performance

Several standard metrics exist for measuring assembly performance in the absence of a reference genome. They include the length of the largest contig, the total length of all contigs, and N50 (which is equal to the longest contig length such that the sum of the lengths of all longer contigs is half the total output assembly). An additional metric is NG50 (Earl *et al.*, 2011), which is similar to N50 but more comparable across assembly algorithms. When the genome length is known, then rather than using each algorithm's estimate of the genome size, which can fluctuate widely depending on the threshold at which small contigs are output, one can use the true genome size. Thus NG50 is defined as the length of the longest contig such that the sum of all longer contigs is half the total genome size. We considered the above-mentioned metrics in our study.

To investigate assembly accuracy, we mapped each contig to the reference genome using NUCmer from the MUMMER package (Delcher *et al.*, 2002). For each contig, we determined to which telomere it maps best according to the total number of aligned bases. The number of aligned bases in each contig forms a more useful foundation for accuracy-informed continuity statistics than the direct number of bases in each contig. Therefore, we also computed the aforementioned metrics using these aligned lengths.

The results of our study for simulated data are summarized in Table 1, while the results for the real data are shown in Table 2. These results are for the 32 telomeric regions, each of length 40 kb. As the tables show, Telescoper exhibited the best performance under most metrics, with notable margins from the second best method. As shown in Table 1, reducing the read length from 101 to 50 bp while keeping all other parameters the same worsened the performance of most algorithms, with ABySS being the most affected.

**Table 1.** Summary of assembly results based on simulated data from 32 telomeric regions each of length 40 kb. ‘%Aligned’ is the ratio of Total Aligned to Total Produced, while ‘%Covered’ is the fraction of the telomeric regions covered by contigs

Results for simulated data D1 (read length = 101 bp)										
	Produced (kb)				Aligned (kb)					
Assembler	N50	NG50	Max	Total	N50	NG50	Max	Total	%Aligned	%Covered
Telescoper	40.0	40.0	41.0	1208	40.0	40.0	40.0	1172	97.0	90.4
ABySS	31.0	31.0	39.0	1296	31.8	31.8	39.3	1244	95.9	84.7
ALLPATHS2	35.2	33.0	39.0	1047	35.2	33.4	40.0	1032	98.5	80.6
SOAPdenovo	25.0	24.0	39.0	1149	28.6	24.6	40.0	1068	92.9	82.3
Velvet	13.9	9.0	31.0	964	13.9	9.5	31.6	947	98.2	73.7
SGA	31.2	27.0	39.0	1110	31.6	27.2	40.0	1075	96.8	82.0

Results for simulated data D2 (read length = 50 bp)										
	Produced (kb)				Aligned (kb)					
Assembler	N50	NG50	Max	Total	N50	NG50	Max	Total	%Aligned	%Covered
Telescoper	39.0	38.0	39.0	1162	38.8	38.3	39.8	1155	99.4	90.3
ABySS	12.1	8.0	31.0	1097	13.7	8.9	31.6	966	88.0	75.0
ALLPATHS2	32.0	27.0	39.0	968	32.8	27.7	40.0	950	98.2	74.3
SOAPdenovo	25.0	21.0	39.0	988	24.6	20.8	40.0	954	96.5	74.3
Velvet	14.0	9.0	31.0	955	14.2	9.5	31.9	939	98.3	73.2

**Table 2.** Summary of results for real data D3. The contigs produced by each algorithm were aligned to the 32 telomeric regions each of length 40 kb. As before, ‘%Covered’ is the fraction of the telomeric regions covered by contigs

	Aligned (kb)				
Assembler	N50	NG50	Max	Total	%Covered
Telescoper	34.5	32.8	39.2	980	75.8
ABySS	12.0	8.3	31.3	971	75.3
ALLPATHS2	26.3	16.5	40.0	923	70.1
SOAPdenovo	21.4	16.2	39.3	879	68.6
Velvet	11.8	6.9	31.3	928	72.2

**Fig. 5.**
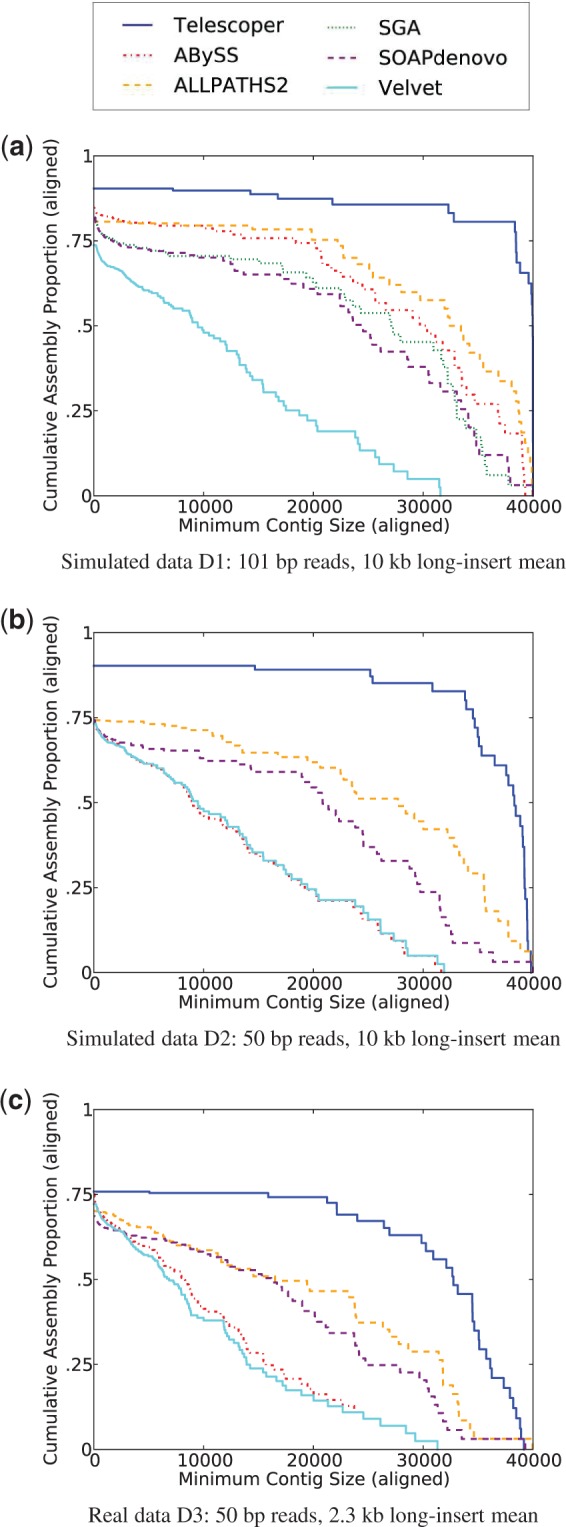
The cumulative proportion of all aligned contigs exceeding the contig size indicated on the *x*-axis. These plots illustrate the continuity and completeness of different assemblies. For any given minimum contig length, Telescoper produced more aligned bases. NG50 can be read from this graph as the *x*-coordinates at which each curve hits the 50% mark of bases output relative to the reference. (**a**) Results on simulated data D1. (**b**) Results on simulated data D2. (**c**) Results on real data D3

Figure 5 provides a more detailed picture of contig length distribution. These plots show the cumulative proportion for all aligned contigs exceeding the contig size indicated on the *x*-axis. NG50 can be read from the plots as the *x*-coordinates at which each curve hits the 50% mark of bases output relative to the reference. The best possible curve is the constant function *y* = 1, so the closer a curve is to that line, the better the performance. Note that for any given minimum contig size (the *x*-axis value), Telescoper produced more alignable bases than all other methods compared, for all three datasets. Furthermore, Figures 5a and b illustrate that Telescoper is more robust to a decrease in read length than are the other algorithms.

For Telescoper, the observed difference between the corresponding curve in Figure 5b and that in Figure 5c is largely attributable to the difference in the insert-size distribution. On simulated 50 bp data with long inserts with mean length 2.2 kb and short inserts with mean length 400 bp, the performance of Telescoper was similar to that shown in Figure 5c (see Supplementary Material), suggesting that Telescoper is robust the complications of real data and that the observed good performance of Telescoper in Figure 5b is due to its improved ability to take advantage of a longer (10 kb instead of 2.2 kb) long-insert distribution.

Of further importance is the extent to which an algorithm produces false bases or contigs. Because we forced each contig to align to a single telomere, chimeric contigs created by joining portions of different telomeres were penalized as having bases that do not align. As shown in the ‘% Aligned’ column of Table 1, Telescoper was the top performer in this regard for D2, and followed ALLPATHS 2 and Velvet closely for D1.

**Fig. 6.**
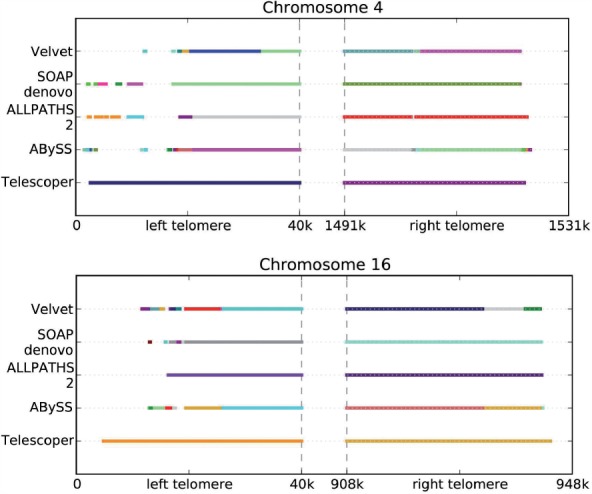
Contig continuity results for real data D3. The left and right telomeric regions (separated by the dotted line) for two different chromosomes are shown, with the aligned contigs displayed for each assembly algorithm. Different colours represent different contigs in the produced assembly, so more colours per method indicates a larger number of contigs. For each telomeric region shown, Telescoper produced a single contig for almost the entire region, while other algorithms often produced many small contigs

Finally, we considered visually examining the alignments of contigs onto each telomeric region. Figure 6 shows the results for two chromosomes, with contigs from each assembly algorithm aligned to them. For each algorithm, each contig is represented by a different colour, so more colours per method indicates a larger number of contigs. For each telomeric region shown, Telescoper produced a single contig for almost the entire region, while other algorithms often produced many small contigs.

## 5 CONCLUSION

We have introduced several new ideas for *de novo* genome assembly, geared towards highly repetitive regions. Our preliminary assembler, Telescoper, proceeds by iteratively extending paths and selecting between them using the empirical distributions formed by both long-insert and short-insert paired-end reads.

The utility of Telescoper was validated in a study on both real and simulated data from the 40 kb telomeric regions of each chromosome of *S. cerevisiae*. For all three datasets tested, Telescoper produced more continuous assemblies than the other algorithms considered. In our evaluations, we tried to include the strongest and most popular algorithms with available implementation. Unfortunately, ALLPATHS-LG (Gnerre *et al.*, 2011) could not be included, because of its small-fragment library requirement mentioned in Section 1. We considered several standard metrics for comparing assemblies, but we note that the task of comparing genome assemblies is a large one, with several papers exclusively devoted to it (Earl *et al.*, 2011; Salzberg *et al.*, 2012).

Other researchers are currently working on algorithms for identifying assembly errors using features derived from read mapping. Rather than having this be a downstream process, we believe that it would help to incorporate such features directly into an assembly algorithm. Here, we make an effort in this direction by scoring assembly extensions according to read-mapping statistics. Although the scoring scheme used in this article may not be optimal, we have demonstrated that the idea of simultaneously pursuing multiple extensions, and concurrently using multiple libraries to score and select among them is promising.

The current implementation of Telescoper can be used as a finishing algorithm to extend contigs into repetitive regions and produce better assemblies for telomeres. Other applications include targeted *de novo* assembly of structural variants and highly variant regions such as human leukocyte antigen. Future work will include extending the ideas presented here to whole-genome assembly, improving error correction, producing more exhaustive listings of potential paths and more thorough evaluation of the alternate paths. Also, additional validation metrics such as those explored by Salzberg *et al.* (2012) can be incorporated as well.

We often see cases where, if we took the union of all assemblies, we could produce a much better final product. This suggests that assembly is not a solved problem, and that the strengths of different algorithms can potentially be combined to produce better assemblies. We believe the ideas behind Telescoper have the potential to improve *de novo* assembly significantly and provide a comprehensive picture of previously unresolved repetitive regions.
